# Early Cardiac Involvement in Treatment-Naïve, Autoantibody-Seropositive Patients with Autoimmune Rheumatic Diseases in the Prodromal Phase—A Cardiovascular Magnetic Resonance Study

**DOI:** 10.3390/jcm15114279

**Published:** 2026-06-01

**Authors:** George Markousis-Mavrogenis, Vasiliki Koulouri, Clio P. Mavragani, Sophie I. Mavrogeni

**Affiliations:** 1 University Research Institute of Maternal and Child Health and Precision Medicine and UNESCO Chair in Adolescent Health Care, National and Kapodistrian University of Athens, 11527 Athens, Greece; georgemm32@gmail.com; 2 Department of Physiology and Pathophysiology, School of Medicine, National and Kapodistrian University of Athens, 11527 Athens, Greece; villykoulouri@gmail.com (V.K.); cliopmavragani@gmail.com (C.P.M.); 3 Olympic Diagnostic/Research Center, 18543 Piraeus, Greece

**Keywords:** cardiovascular magnetic resonance, inflammation, fibrosis, myocarditis, edema, overlap syndrome, undifferentiated autoimmune disease

## Abstract

**Background:** Autoimmune rheumatic diseases (ARDs) often present diagnostic challenges, particularly in undifferentiated disease or overlap syndromes. Autoantibodies (AABs) serve as early biomarkers, but their relationship with cardiac involvement during the prodromal phase remains unclear. We hypothesized that cardiac involvement is an early, unifying feature in AAB-seropositive patients with suspected ARD/overlap syndromes but an as-of-yet unclear diagnosis. **Methods:** We prospectively recruited 18 treatment-naïve patients (mean age 52 ± 17 years, 94.4% women) with suspected undifferentiated ARD/overlap syndromes who were seropositive for myositis-specific (MSAs), myositis-associated (MAAs), or scleroderma-specific autoantibodies (SScSAs). All underwent comprehensive rheumatologic, pulmonologic, and cardiac evaluations, including multiparametric cardiovascular magnetic resonance (CMR) to assess myocardial inflammation, edema, and fibrosis. **Results:** Despite normal echocardiograms, electrocardiograms, and inflammatory biomarkers, all patients exhibited CMR evidence of cardiac involvement. Active myocardial inflammation (revised Lake Louise criteria) was confirmed in 66.7%, while subepicardial fibrosis was universal (median 5.0% of LV mass). During the 12-month follow-up, all patients with evidence of inflammation received immunosuppressive and cardioprotective therapy, leading to symptomatic improvement in all and reduced inflammation in 75% of repeat CMRs (3/4 patients). A definitive rheumatologic diagnosis was established in all cases, with 50% classified as overlap syndromes. **Conclusions:** Cardiac involvement is a highly prevalent disease manifestation in AAB-seropositive patients with suspected ARD/overlap syndromes and can be detected by CMR during the prodromal phase, even before diagnostic criteria are met. These findings support early CMR integration in the workup of such patients to guide timely immunosuppressive and cardioprotective interventions.

## 1. Introduction

Autoimmune rheumatic diseases (ARDs) represent a cluster of syndromes with the common feature being the loss of tolerance to self-antigens, eventually leading to the generation of inappropriate autoimmune responses. The exact differentiation between various subtypes of ARDs depends on established diagnostic criteria, including signs/symptoms, disease manifestations, and laboratory measurements [1]. However, this process is not always straightforward, particularly in cases where patients may either only partially fulfill the diagnostic criteria for a specific ARD, termed undifferentiated ARDs, or may fulfill the criteria of multiple distinct ARDs at the same time, termed autoimmune overlap syndromes [2,3,4]. The most well-defined overlap syndrome is mixed connective tissue disease (MCTD), although other syndromes, including rheumatoid arthritis (RA), systemic lupus erythematosus (SLE), systemic sclerosis (SSc), and idiopathic inflammatory myopathies (IIM), may be involved [2,3,4]. Importantly, overlap syndromes are less common than the conditions they encompass, and thus, significant delays can be observed from initial symptom onset to definitive diagnosis [5].

Autoantibody (AAB) profiling represents the “immunologic fingerprint” of ARDs, and AAB seropositivity can often be detected years before disease onset in a significant subset of patients with ARDs. In the prodromal phase of disease, where clinical manifestations may be vague, AABs can be valuable tools for the early detection, accurate classification and implementation of tailored diagnostic and therapeutic strategies [6]. Furthermore, several AABs are linked to specific organ involvement and can also provide significant prognostic information [7,8,9,10]. Particularly in the case of autoimmune overlap syndromes, myositis-specific autoantibodies (MSAs) (against TIF1γ, NXP2, MDA5 and several t-RNA synthetases), myositis-associated autoantibodies (MAAs) (PM/Scl-100, Ku), and scleroderma-specific autoantibodies (SScSAs) (anti-centromere antibodies, topoisomerase I, RNA polymerase III) may additionally aid in the diagnosis.

Nevertheless, even though the presence of AABs can support the diagnosis of an ARD, a definitive diagnosis also requires a corresponding clinical phenotype and/or organ involvement. Using multiparametric cardiovascular magnetic resonance (CMR), we have previously identified occult cardiac involvement as a common disease manifestation in treatment-naïve patients at the time of diagnosis of an ARD [11]. However, it remains unclear whether cardiac involvement may be present even before a definitive diagnosis of an ARD can be made. Furthermore, the relationship between cardiac involvement identified using CMR and seropositivity for AABs has not been extensively investigated, particularly in the prodromal phase of disease. We thus hypothesized cardiac involvement may constitute a common initial disease manifestation in patients with a clinical suspicion but as-of-yet unclear diagnosis of undifferentiated ARD/overlap syndrome, but with the common feature of seropositivity for MSAs, MAAs and/or SScSAs. To investigate this hypothesis, we conducted a prospective cohort study recruiting patients from our specialized rheumatology outpatient clinic.

## 2. Methods

### 2.1. Patients

We prospectively recruited 18 consecutive, treatment-naïve patients (aged ≥18 years) with a clinical suspicion of an undifferentiated ARD/overlap syndrome who did not fulfill any of the diagnostic criteria for the establishment of a specific diagnosis at the time of recruitment. All patients needed to be seropositive for at least one clinically employed AAB and needed to have undergone extensive rheumatologic, pulmonological and cardiac evaluations, including a patient history, physical examination, surface electrocardiogram, echocardiogram, spirometry with diffusion testing and contrast-enhanced CT, without evidence of pathologic findings. The presence of cardiac symptoms was not a prerequisite for study enrollment.

The main presenting symptoms were recorded, and all patients were referred to our specialist cardiovascular imaging center with extensive expertise in cardio-rheumatology for a comprehensive multiparametric CMR examination in order to evaluate the presence of cardiac involvement. Participants with evidence of myocardial inflammation received immunosuppressive treatment (at the discretion of the referring rheumatologist) and cardioprotective treatment (angiotensin receptor antagonists and β-adrenoreceptor blockers) and were followed up clinically and, in some cases, with repeat CMR to evaluate treatment effectiveness. Evidence-based heart failure treatment was initiated in all patients with impaired systolic function, irrespective of the presence of myocardial inflammation. During the 12-month follow-up period, we recorded whether a rheumatologic diagnosis could be established using currently employed diagnostic criteria. Patients were excluded from the study if aged <18 years or if any of the common contraindications to CMR were present (presence of metallic clips and non-MRI compatible devices).

Written informed consent was obtained from all patients before inclusion in the study. The study was conducted according to the principles outlined in the Declaration of Helsinki, and the study was approved by the Olympic Diagnostic/Research Center ethics committee (0106/10 January 2025).

### 2.2. Cardiovascular Magnetic Resonance

#### 2.2.1. CMR Protocol

All study participants were evaluated using a 3.0 T scanner (Magneto Skyra Fit, Siemens Healthineers, Erlangen, Germany). The CMR protocol included the evaluation of biventricular function and dimensions, left ventricular mass, and tissue characterization using edema, inflammation, and fibrosis evaluation. A gadolinium-based contrast agent was not administered in 3/18 patients due to known history of severe allergic reaction. Cut-off points for normal values of the CMR investigation were based on the 99th percentile of values obtained from fifty healthy volunteers examined in our unit.

#### 2.2.2. Functional Analysis

The CMR examination included standard functional imaging using a breath-held balanced steady state precession (bSSFP) cine sequence [12,13] with the following acquisition parameters: 58° flip angle, rate-3 parallel imaging, matrix size 256 × 192, pixel size 1.6 mm × 1.6 mm, slice thickness 6 mm, BW 977 Hz/Px, TE/TR 1.4 ms/3.3 ms echo spacing and a temporal resolution of 32.5 ms. All cine imaging included the entire LV from base to apex using short-axis slices, according to a protocol that was described in previous studies [11,12].

#### 2.2.3. STIRT2 and T2 Mapping for Edema Imaging

Black-blood short tau inversion recovery T2-weighted images (STIR-T2) were acquired for edema detection. The ratio of myocardial to skeletal muscle STIR-T2 was used to define the presence of edema. For T2 mapping, data were acquired in basal-, mid-ventricular, and apical SAX planes using a previously described protocol [11].

#### 2.2.4. T1 Imaging for Inflammation/Fibrosis Detection

T1-weighted spin-echo early gadolinium enhanced (EGE) and phase-sensitive inversion recovery late gadolinium enhanced (LGE) images were acquired after intravenous injection of gadobenate dimeglumine contrast medium (Gadoteric acid, Cyclolux, VIANEX), as described previously [14].

Myocardial T1 mapping measurements were acquired using a modified Look–Locker inversion recovery (MOLLI) sequence with MOtion Correction (MOCO) in basal, midventricular and apical short-axis (SAX) slices with electrocardiographic gating and breath holding [15]. Pre- and post-contrast T1 mapping images were acquired according to previously described protocols [14].

#### 2.2.5. Post-Processing Analysis

Two expert clinicians (SM, GMM) evaluated the images using the Syngo Siemens protocol. The following functional parameters were calculated: left/right ventricular end systolic volume (LVESV, RVEDV) and end diastolic volume (LVEDV, RVEDV), ejection fraction (LVEF, RVEF), and LV mass (LVM). A normal LVEF was defined as ≥55% [16]. Additionally, the presence or absence of LGE was identified according to the American Heart Association (AHA) 17-segment model [17]. Pre- and post-contrast T1 maps were used together with hematocrit values to calculate an extracellular volume fraction (ECV) map [18]. T1, T2 and ECV mappings were evaluated on the same three LV short-axis slices using the Syngo Siemens protocol.

#### 2.2.6. CMR Image Analysis

Global myocardial inflammation was assessed in STIR-T2 images according to a previously described protocol [19]. The LGE lesions were quantified as % of LV mass by consensus agreement of two experienced observers. Global T1, ECV, and T2 values were calculated according to a previously described protocol [20].

#### 2.2.7. Evaluation Based on the Lake Louise Criteria

Myocardial inflammation was evaluated using the revised Lake Louise criteria [19]. CMR indices were divided into T1-based and T2-based indices. When at least one index was pathologic according to locally validated normal cut-off values, the corresponding criterion was considered positive. When both criteria were positive at the same time, myocardial inflammation is confirmed with high sensitivity [19,20].

### 2.3. Statistical Analyses and Data Visualization

All statistical analyses and data visualization were carried out using R-Studio (R version 4.2.0). Normally distributed variables are presented as mean (standard deviation), non-normally distributed continuous variables are presented as median (interquartile range) and binary/categorical variables are presented as number (percentage).

## 3. Results

**Figure 1:**
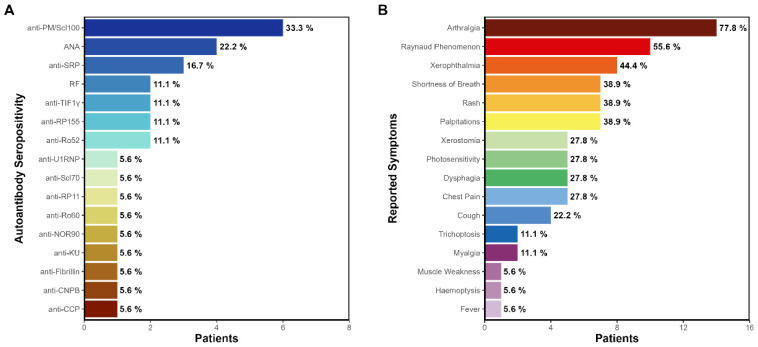
(**A**) Proportion of patients that was seropositive for at least one of the measured autoantibodies. (**B**) Frequency of reported presenting symptoms at study inclusion.

The study population included 18 participants [17 (94.4%) women] with a mean age of 52 (17) years. The frequency of seropositivity for various AABs is presented in Figure 1A. The most frequently observed AABs were anti-PM/Scl100 with six (33.3%) seropositive participants, ANA with four (22.2%) seropositive participants and anti-SRP with three (16.7%) seropositive participants. The relative frequency of presenting symptoms is illustrated in Figure 1B. Regarding cardiac symptoms, the most frequent were shortness of breath and palpitations, reported by seven (38.9%) participants, while five (27.8%) participants reported chest pain. A total of five (27.8%) participants did not report any cardiac symptoms. A complete blood count and metabolic panel, including C-reactive protein levels at study inclusion, showed no abnormalities in any of the participants (Table 1).

The evaluation of biventricular systolic function demonstrated that three (16.7%) and two (11.1%) participants, respectively, showed reduced LV and RV systolic function. All study participants showed CMR evidence of myocardial involvement. Based on the Lake Louise criteria, all participants had a positive T1-criterion, and 12 (66.7%) had a positive T2-criterion. As such, the presence of active myocardial inflammation could be confirmed with high sensitivity in 12 (66.7%) participants. The remaining participants who did not have evidence of active myocardial inflammation (no presence of edema) showed evidence of previous myocardial inflammation with subepicardial non-ischemic myocardial fibrosis and increased T1 mapping or ECV values. Subepicardial LGE was identified in all participants, and its extent ranged from 1% to 15% of LV mass [median 5.0%, IQR (1.0–10.0%)]. Detailed CMR findings are presented in Table 1.

In all patients with evidence of recent inflammation along with other autoimmune features; immunosuppressive treatment, including corticosteroids, methotrexate, and hydroxychloroquine; and cardioprotective treatment, including angiotensin converting enzyme inhibitors/angiotensin receptor blockers and β-blockers, was initiated. Evidence-based heart failure therapy was initiated in three patients with LV systolic dysfunction (LVEF < 55%), irrespective of the presence of inflammation.

**Figure 2:**
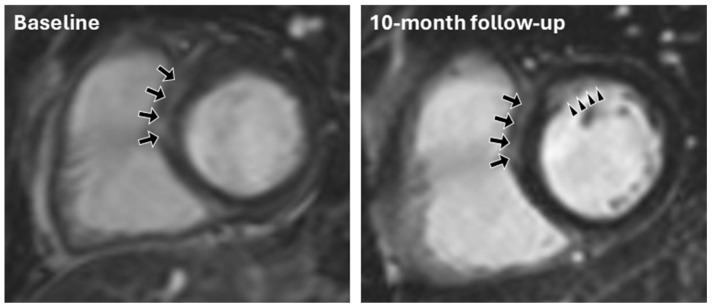
Sort axis midventricular late gadolinium enhanced (LGE) images of a patient with autoimmune overlap syndrome (systemic sclerosis–Sjögren syndrome overlap) at study inclusion (**left**) and at 10-month follow-up following an episode of chest pain with troponin elevation (**right**). The patient showed evidence of a subepicardial scar in the interventricular septum (arrows) at baseline and showed a new subendocardial scar (arrowheads) following the chest pain episode. No obstructive epicardial coronary artery disease was detected (see also Figure 3).

**Figure 3:**
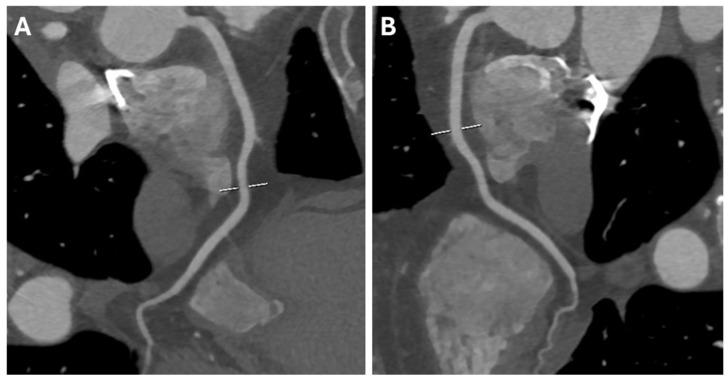
Computed tomography coronary angiography of the same patient discussed in Figure 2, showing a non-obstructed left anterior descending artery (**A**) and right coronary artery (**B**) after an episode of chest pain with troponin elevation.

During 12 months of clinical follow-up, all patients with cardiac symptoms reported a symptomatic improvement. Repeat CMR examinations were available in 4/18 patients, and improvement in myocardial inflammation was seen in 3/4 (75%) patients. The remaining one participant developed chest pain at 10 months following the first CMR evaluation. Troponin values were increased, and follow-up CMR revealed a new subendocardial myocardial infarction (Figure 2). This was deemed to be due to microvascular coronary artery disease, as a computed tomography coronary angiography showed non-obstructed epicardial coronary arteries (Figure 3).

All rheumatologic symptoms improved post-immunosuppressive treatment. Within the 12-month follow-up period, a rheumatologic diagnosis could be established in all patients based on currently employed diagnostic criteria. The final rheumatologic diagnoses are presented in detail in Table 1 and included a diagnosis of overlap syndrome in nine (50%) patients, undifferentiated connective tissue disease (UCTD) in three patients (16.7%), Sjögren syndrome in three (16.7%) patients, and SSc, dermatomyositis and myositis, each in one (5.6%) patient.

## 4. Discussion

In this prospective cohort study of treatment-naïve and AAB-seropositive patients with suspected ARD but an as-of-yet unclear diagnosis, we demonstrate that cardiac involvement is universally present, even in the absence of a definitive diagnosis or cardiac symptoms. Using multiparametric CMR, we identified evidence of active myocardial inflammation in 66% of patients, while subepicardial fibrosis was detected in all cases. Notably, echocardiographic and electrocardiographic evaluation, as well as inflammatory biomarkers, were within normal limits in all patients. A rheumatologic diagnosis could be established in all patients within the 12-month follow-up period after study inclusion. Collectively, our findings support the notion of a prodromal phase in autoimmune diseases, where seroconversion may predate clinical diagnosis by months to years [21].

In previous work, we have demonstrated using CMR that cardiac involvement is common in patients with treatment-naïve ARDs at the time of diagnosis [11], while other investigators have also reported the presence of early subclinical abnormalities in vascular and myocardial function in newly diagnosed, treatment-naïve patients with RA [22]. Furthermore, we have previously described the presence of myocardial fibrosis and perfusion defects in patients with established MCTD who were seropositive for anti-RNP AABs [23]. To our knowledge, this is the first study to systematically evaluate cardiac involvement in the pre-diagnostic phase of suspected ARD/overlap syndromes, before patients meet diagnostic criteria for a specific syndrome.

### 4.1. Clinical Implications

The findings of our study have important clinical implications. Firstly, we demonstrate that irrespective of the final rheumatologic diagnosis, cardiac involvement represents a unifying disease manifestation in treatment-naïve patients with undifferentiated ARD/overlap syndrome. Perhaps more strikingly, the entire patient cohort did not demonstrate other abnormalities in cardiopulmonary workup (including lung CT, echocardiography and electrocardiography), while biochemical indices of inflammation were not pathologically elevated. Recent literature also suggests that patients with myocarditis and regional LV involvement can have normal CRP levels [24]. Thus, the evaluation of cardiac involvement should not cease when a standard workup shows no pathologic findings. Instead, multiparametric CMR should be considered as part of the routine workup for patients with seropositivity for MSAs, MAAs, or SScSAs, even in the absence of cardiac symptoms. The role of CMR, especially in the diagnosis of myocarditis, is particularly emphasized in recent ESC Guidelines [25]. A consensus-based decision algorithm for when a CMR study could be considered in patients with established ARDs, together with a standardized study protocol have also been previously published [20]. Nevertheless, specific recommendations for patients with undifferentiated diseases are still pending.

Secondly, the detection of cardiac involvement is vital in the diagnosis of ARDs and constitutes an actionable clinical phenotype that may motivate the initiation of disease-modifying and cardioprotective treatment [26,27,28,29]. In our study, this approach led to symptomatic improvement in all patients with cardiac symptoms and a reduction in myocardial inflammation in 75% of those who underwent repeat CMR. Similar approaches incorporating imaging follow-up of disease-modifying treatment using CMR have been successfully used to monitor response to treatment in patients with an established diagnosis of SSc and primary heart involvement [30].

Lastly, the case of a patient who developed a subendocardial myocardial infarction (MINOCA) with non-obstructed coronaries highlights the potential pathophysiologic role of microvascular dysfunction in ARD/overlap syndromes. MINOCA is increasingly recognized in ARDs and is thought to result from endothelial dysfunction, vasculitis, or microthrombosis [31,32].

### 4.2. Autoantibody Profiling and Cardiac Involvement

The autoantibody profile of our cohort may provide insights into the pathophysiological mechanisms underlying cardiac involvement. The most frequently detected autoantibodies were anti-PM/Scl100 (33%), ANA (22%), and anti-SRP (17%). Anti-PM/Scl AABs have been reported to be associated with a specific phenotype of SSc with more prevalent involvement of skeletal muscle [33,34]. Our findings suggest that cardiac involvement may also be more prevalent in this patient subgroup. Anti-SRP antibodies are a marker of immune-mediated necrotizing myopathy and have been associated with severe cardiac involvement, including dilated cardiomyopathy and heart failure [35]. One study has reported that anti-SRPs induce cardiac diastolic dysfunction through oxidative stress, although other pathomechanisms still remain to be elucidated [36]. Furthermore, ANAs have been associated with cardiovascular events both in patients with and without ARDs and have been shown to be present in patients with infarct-like myocarditis on CMR [37,38].

Less frequent AABs in our cohort included anti-TIF1-γ, anti-RP155, anti-Ro52, and RF (11% of patients in each case). Although no association between anti-TIF1-γ or anti-RP155 and cardiac involvement has previously been reported, anti-Ro52 seropositivity has been linked to cardiac conduction disorders and arrhythmias in a large population-based study [39], while RF is associated with cardiovascular events [37]. Collectively, the presence of the aforementioned, as well as other AABs, in our cohort suggests that serological profiles may not only aid in diagnosis but should also motivate additional investigation for cardiac involvement. However, our investigation was limited to clinically used AABs, and future investigations should include emerging AABs targeting cardiac antigens, which seem to be associated with a high probability of cardiac involvement in patients with SSc [40].

### 4.3. Limitations

This study has a number of important limitations. Firstly, the patient population was small and from a single center, making generalization of our findings difficult. Follow-up was limited to 12 months, and thus, no conclusions regarding long-term outcomes can be drawn. Additionally, given the specialization of our referral center in ARDs, the study population may have been subject to a higher pre-test probability of cardiac involvement, as well as a selection bias. Furthermore, the lack of CMR evaluation post-treatment in the majority of patients did not permit a comprehensive serial evaluation of initially identified abnormalities after treatment initiation. Contrast agent administration was not possible in all patients, thus limiting the number of contrast-enhanced studies. Lastly, the initial standardized echocardiographic evaluation did not include ventricular strain analysis, which could potentially reveal actionable myocardial abnormalities.

## 5. Conclusions

In conclusion, our study demonstrates that cardiac involvement is a common and early disease manifestation in patients with seropositivity for MSAs, MAAs, or SScSAs and a clinical suspicion of ARD/overlap syndrome, despite an otherwise unremarkable workup. Using multiparametric CMR, we identified active or past myocardial inflammation and fibrosis in the prodromal phase of ARD, which could be definitively established within 12 months following the CMR examination. These findings highlight the potential of CMR as a tool for early detection and support the early initiation of immunosuppressive and cardioprotective therapies. Future research should focus on validating these findings in larger cohorts, as well as evaluating other emerging AABs, like those targeting cardiac antigens.

## Figures and Tables

**Table 1:** Descriptive statistics of the 18 participants in the study.

Variables	Descriptive Statistics
**Cohort Size**	18
**Demographics**	
Age (Years)	52 (17)
Female Sex	17 (94.4%)
**Final Rheumatologic Diagnosis**	
DM	1 (5.6%)
Myositis	1 (5.6%)
SSc	1 (5.6%)
Sjögren Syndrome	3 (16.7%)
UCTD	3 (16.7%)
**Overlap Syndromes (Total)**	9 (50.0%)
MCTD	2 (11.1%)
SLE-Overlap	1 (5.6%)
RA-MCTD Overlap	1 (5.6%)
RA-SSc overlap	1 (5.6%)
SS-Myositis Overlap	1 (5.6%)
SS-SSc sine scleroderma Overlap	1 (5.6%)
SS-SSc Overlap	1 (5.6%)
SSc-IIM overlap	1 (5.6%)
**CMR Measurements**	
LVEDV (mL)	131 (29)
LVESV (mL)	53 (15)
LVEF (%)	60 (7)
RVEDV (mL)	131 [105, 149]
RVESV (mL)	58 (29)
RVEF (%)	55 (10)
EGE	4.1 [1.4, 8.5]
LGE (%LV Mass)	5.0 [1.0, 10.0]
Native T1-Mapping (ms)	1310 (64)
ECV (%)	29.8 (4.0)
STIR T2 Ratio	1.80 (0.5)
T2-Mapping (ms)	51.6 (5.9)
**Locally Used Normal Cut-off Values for CMR Measurements**	
EGE > 4	8 (53.3%)
LGE > 0% of LV Mass	12 (80.0%)
Native T1-Mapping > 1250 ms	15 (83.3%)
ECV > 28%	9 (60.0%)
STIR T2 Ratio > 1.9	7 (38.9%)
T2-Mapping > 50 ms	9 (50.0%)
LVEF < 55%	3 (16.7%)
RVEF < 50%	2 (11.1%)
**Lake Louise Criteria for Myocardial Inflammation**	
T1-Criterion	18 (100.0%)
T2-Criterion	12 (66.7%)
Both Criteria	12 (66.7%)
**Complete Blood Count**	
WBC (×10^9^/L)	6821 (2870)
Neutrophils (%)	57 (15)
Lymphocytes (%)	35 (13)
Monocytes (%)	6.3 (2.5)
Eosinophils (%)	2.1 (1.6)
Hb (g/dL)	13.2 (1.2)
MCV (fl)	89.6 (3.3)
Platelets (×10^9^/L)	272 (51)
**Metabolic Profile**	
Serum Creatinine (mg/dL)	0.74 (0.12)
ASAT (U/L)	17.5 [14.8, 21.0]
ALAT (U/L)	18.00 [14.3, 25.3]
γGT (U/L)	18.5 [14.5, 26.0]
ALP (U/L)	72.0 [52.0, 92.0]
CK (U/L)	64.0 [41.0, 91.0]
LDH (U/L)	214.6 (85.2)
CRP (mg/L)	0.26 [0.05, 0.43]

DM: dermatomyositis, MCTD: mixed connective tissue disease, SSc: systemic sclerosis, UCTD: undifferentiated connective tissue disease, SLE: systemic lupus erythematosus, RA: rheumatoid arthritis, SS: Sjögren syndrome, IIM: idiopathic inflammatory myopathy, LV/RV: left/right ventricular, EDV/ESV: end-diastolic/end-systolic volume, EF: ejection fraction, EGE/LGE: early/late gadolinium enhancement, CMR: cardiovascular magnetic resonance, WBC: white blood cell count, Hb: hemoglobin, ASAT: aspartate aminotransferase, ALAT: alanine aminotransferase, γGT: γ-glutamyl transferase, ALP: alkaline phosphatase, CK: creatine kinase, LDH: lactate dehydrogenase, CRP: C-reactive protein.

## Data Availability

The original contributions presented in this study are included in the article. Further inquiries can be directed to the corresponding author.
